# Determinants and predictive modeling of long-acting reversible contraceptive use in Sub-Saharan Africa: evidence from DHS data using machine learning and association rule mining

**DOI:** 10.1016/j.xagr.2026.100662

**Published:** 2026-06-12

**Authors:** Befkad Derese Tilahun, Mulat Ayele, Eyob Shitie Lake, Gizachew Yilak, Tegene Atamenta Kitaw, Bekalu Alemayehu Attinafu

**Affiliations:** 1 Department of Nursing, College of Health Science, Woldia University (Tilahun, Yilak, Kitaw and Alemayehu), Woldia, Ethiopia; 2 Department of Midwifery, College of Health Science, Woldia University (Ayele and Lake), Woldia, Ethiopia

**Keywords:** LARC, machine learning, reproductive health, Sub-Saharan Africa

## Abstract

**BACKGROUND:**

Long-acting reversible contraceptives (LARCs), including intrauterine devices and implants, are highly effective in preventing unintended pregnancies. Despite their benefits, utilization remains low across many Sub-Saharan African (SSA) countries.

**OBJECTIVE:**

This study aimed to predict LARC utilization and identify key determinants among women of reproductive age in SSA using advanced machine learning techniques.

**METHODS:**

A secondary analysis was conducted using the latest Demographic and Health Survey (DHS) datasets from eight SSA countries, yielding a weighted sample of 29,016 women aged 15–49 years. Data preprocessing included cleaning, feature engineering, variable selection, and class balancing with SMOTE. Twelve machine learning models were developed, and the best-performing model was optimized using Bayesian methods. Association rule mining (Apriori algorithm) was applied to uncover hidden patterns among predictors.

**RESULTS:**

The pooled prevalence of LARC use was 29% (95% CI: 21%–38%), with high between-country heterogeneity (I²=99.67%). Random Forest achieved the best performance after optimization, with an accuracy of 87.1%, AUC of 81.0%, and F1 score of 85.0%. Major predictors included country, education, parity, marital status, and age. Association rule mining showed that rural, uneducated, poor, and married women in Senegal and Burkina Faso had a higher likelihood of LARC use (Lift =2.25).

**CONCLUSION:**

Machine learning identifies potential predictors of LARC utilization and identifies key determinants in SSA. Targeted interventions focusing on rural, low-income, and low-education groups may improve LARC uptake and reduce unmet family planning needs.

**AJOG Global Reports at a Glance**

**Why was the study conducted?**

To identify determinants of long-acting reversible contraceptive (LARC) utilization and develop predictive models among reproductive-age women in Sub-Saharan Africa using Demographic and Health Survey (DHS) data and machine learning techniques.

**Key findings**

The pooled prevalence of LARC use was 29%. Random Forest showed the best predictive performance (accuracy 87.1%, AUC 81.0%). Major predictors included country, education, parity, marital status, and age. Association rule mining identified higher LARC use among rural, poor, married, and uneducated women in Senegal and Burkina Faso.

**What does this study add to what is known?**

This study demonstrates the utility of machine learning and association rule mining in predicting LARC utilization across multiple Sub-Saharan African countries and highlights important contextual and demographic disparities that can inform targeted family planning interventions.

## Introduction

Contraception is a vital tool in reproductive health, primarily used to reduce the likelihood of pregnancy following sexual intercourse.^1^ It plays a crucial role for women of reproductive age in determining the number, timing, and spacing of children and helping to prevent unintended pregnancies. Contraception also allows adequate time for a woman to recover from the physical demands of previous pregnancies.^2^ As a component of maternal health services, contraceptive care contributes significantly to reducing maternal, infant, and child mortality and to enhancing the overall health and well-being of mothers.^3^

Contraceptive methods are broadly categorized into traditional and modern methods. Modern contraception is further divided into short-acting and long-acting methods. Among these, long-acting contraceptive (LAC) methods are recognized as highly effective and suitable options for many women.^4^ Long-acting contraceptives can be either reversible or permanent. Reversible methods include hormonal contraceptive implants, which release progestin to prevent pregnancy for 3–5 years, and intrauterine contraceptive devices (IUCDs), which are nonhormonal devices placed in the uterus, offering protection for up to 12 years. Permanent methods such as vasectomy and tubal ligation provide lifelong pregnancy prevention.^5^

Despite their effectiveness, the global uptake of Long-Acting Reversible Contraceptives (LARCs) remains low. Approximately 44% of women worldwide do not use long-acting and permanent contraceptive methods.^6^ In Africa, usage is particularly limited, with only 4.6% of women using implants and just 1% using IUCDs. Most contraceptive users in the region rely on short-acting methods, and 86% of women of reproductive age do not use IUCDs at all.^6^

Around 214 million women in developing countries lack access to modern contraceptive methods, leading to nearly half of all pregnancies being unintended and one-third of which result from contraceptive failure. Underuse of contraception contributes to high fertility rates and elevated maternal mortality.^7^ Long-acting contraceptive methods have many advantages compared to other contraceptive methods; they are convenient, effective, long-lasting, reversible, and cost-effective. In addition to these, they are not dependent on compliance with taking the oral contraceptives daily or taking the regular injection at clinics.^8^

Beyond preventing pregnancy, contraceptives offer additional health benefits, including menstrual cycle regulation, alleviation of premenstrual mood disturbances, and a reduced risk of endometrial cancer.^7^ Unintended pregnancies, particularly among young girls, carry severe physical and psychological consequences and impose significant social and economic burdens.^9^ Family planning (FP) is crucial for reducing maternal and child mortality, preventing unintended pregnancies, and enhancing the health and well-being of women and their families. It also facilitates adequate spacing between pregnancies, which is especially critical for younger women who are at higher risk of complications.^10^

Contraceptive discontinuation undermines the effectiveness of FP programs and contributes to undesired fertility. This issue can be addressed by expanding the range of available methods and enhancing service quality through improved client education, enhanced provider competence, effective interpersonal communication, robust follow-up mechanisms, and seamless service continuity.^11^ Despite advancements in FP programs, about 38% of users globally discontinue their chosen contraceptive method within the first year of use.^12^

The consequences are alarming: approximately 282 million unintended pregnancies and 40 million unsafe abortions occur globally each year, with an estimated 30 million women suffering from abortion-related complications and 5–10 million women aged 15–49 dying as a result.^13^ The consequences of not using modern family planning are a decline in economic status, reduced education, a lack of quality of life, and increased maternal and perinatal mortality.^14^ Around 15% of reproductive-age women use long-acting reversible contraceptive methods; however, only 3% of women in sub-Saharan Africa use long-acting reversible contraceptive methods regularly.^15^

Machine learning (ML), a subfield of artificial intelligence, offers promising opportunities to strengthen healthcare systems. ML can analyze large and complex datasets to uncover hidden patterns and build predictive models.^16^ This study applies a machine learning-based approach to predict the likelihood of long-acting reversible contraceptive utilization and identify the key factors influencing women’s choices.

### Study setting

The research was undertaken in Sub-Saharan Africa, a region with a rapidly increasing and diverse population. Current estimates place the population at around 1.29 billion people.^17^ The area is divided into four major geographical regions: Eastern, Central, Western, and Southern Africa, each with distinct cultural, social, and economic contexts. This study utilized data from the recently released Phase VIII Demographic and Health Surveys (DHS), specifically drawing on data from eight Sub-Saharan African countries: Burkina Faso, Côte d’Ivoire, Ghana, Kenya, Lesotho, Mozambique, Tanzania, and Senegal.

### Population and eligibility criteria

This study included reproductive-age (15–49 years) women who had used long-acting reversible contraceptives, either IUCD or an implant, in the selected enumeration areas at the time of DHS data collection.

### Data source

This study utilized secondary data from the most recent Demographic and Health Surveys (DHS) conducted in 8 sub-Saharan African countries. DHS datasets were obtained from the DHS Program website (http://www.dhsprogram.com), following the submission of the study's justification and project title and after permission was granted. The Women's Record (IR) dataset was used for this investigation. A two-stage probability sampling method, stratified by geographic region and urban/rural areas within each region, was applied to select study participants, ensuring the sample fully represents the target population of Sub-Saharan African countries. After data preparation, a weighted sample of 29,016 reproductive-age women was included in the study.

### Sample size and sampling procedure

This study used a weighted sample of 29,016 reproductive-age women. Due to the nonproportional distribution of the sample size across different regions, variations between urban and rural areas, and potential differences in response rates, sampling weights were applied to maintain representativeness. Participants were selected using a two-stage stratified cluster sampling procedure. In the first stage, Enumeration Areas (EAs) were randomly selected based on their clusters. A stratified sample of census EAs from both urban and rural areas was chosen with complete household listings, using systematic probability sampling. This sampling was based on a sampling frame that contained population and household information from the Population and Housing Census (PHC). In the second stage, households within the selected EAs were chosen using equal probability systematic sampling. In each selected household, reproductive-aged women were interviewed using an individual questionnaire.^18^

### Study variables and measurements

#### Dependent variable

The dependent variable in this study was the utilization of long-acting reversible contraceptives (LARCs) among reproductive-aged women, specifically focusing on intrauterine devices (IUCDs) and implants. It was dichotomized as follows: “Yes” =1, indicating that a woman had used at least one of these methods within the five years preceding the survey, and “No” =0, indicating that she had not used either method during that period.

#### Independent variables

The study incorporated a broad set of independent variables categorized into five groups. Socio-demographic and economic factors included age, education level, marital status, and household wealth level. Living conditions and environmental factors covered the place of residence, while family and reproductive factors included family size, birth order, and birth interval. Finally, access to media and information was measured through mass media exposure.

#### Operational definition

The dependent variable was the current use of long-acting reversible contraceptives (LARCs), specifically intrauterine devices (IUCDs) and implants, among women of reproductive age. It was dichotomized as “Yes” (coded as 1) if a woman was currently using at least one of these methods at the time of the survey, and “No” (coded as 0) if she was not using either method.

#### Data analysis procedure

This study employed Demographic and Health Survey (DHS) data from sub-Saharan Africa (SSA) to predict the utilization of long-acting reversible contraceptives (IUCDs and implants) and to identify its determinants among women of reproductive age. We used the R programming language and several essential packages, including dplyr, caret, gbm, pROC, DMwR, and ggplot2, for data cleaning, feature engineering, model development, model validation, and visualization. Both traditional machine learning algorithms and resampling techniques, such as SMOTE, were applied to address class imbalance and improve predictive accuracy. The resulting model not only forecasts the likelihood of long-acting contraceptive use but also identifies key socio-demographic and reproductive factors influencing utilization, offering valuable insights for program planning and policy interventions.

#### Data preprocessing

**Figure 1:**
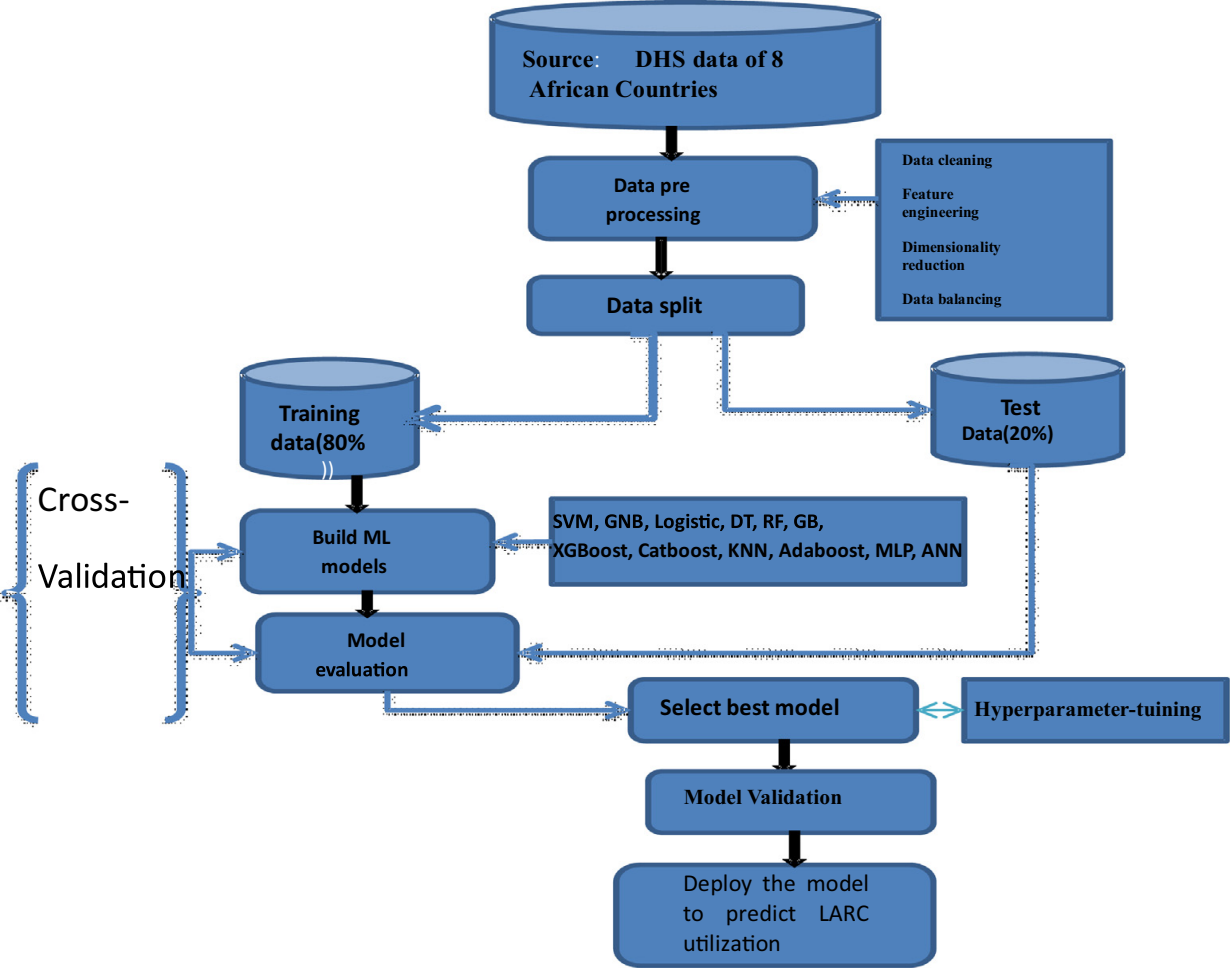
Study work flow diagram of long-acting reversible contraceptive (LARC) utilization across selected African countries

Data processing is a machine learning technique that transforms raw data into a comprehensible format.^19^ In this study, we employed the major data preprocessing steps, which included dimensionality reduction, data transformation, data discretization, data integration, and data cleaning. The advantages of data preprocessing include improving model accuracy through tasks such as data cleaning, exploratory data analysis, normalization, dimensionality reduction, data transformation, and data integration, all of which can positively impact the model's performance^20^ Figure 1.

Tilahun et al. Determinants and predictive modeling of long-acting reversible contraceptive use in Sub-Saharan Africa. Am J Obstet Gynecol 2026.

#### Data cleaning

Our data transformation journey began with the powerful R programming language (v4.5, www.r-project.org), where we built a rigorous preprocessing pipeline designed to extract every ounce of quality from our dataset. In this study, data cleaning was applied to manage outliers, imbalanced outcomes, noise, and missing values. Since raw data often contain gaps that can bias or distort model training and testing, preprocessing was essential.^21^ Among the 29,016 records, nearly 6.6% had fewer than 643 missing values across critical features. Both categorical and continuous variables exhibited missingness, with rates of 4.3% for maternal age at birth, 7.3% for birth order, and 2% for age at first sex. To address this, we applied mean imputation for continuous variables and mode imputation for categorical variables. Outliers, defined as data points that deviate substantially from the distribution of other observations, were detected using visualization techniques such as box plots and chi-square tests. For removal, we employed the Variance Inflation Factor (VIF) scoring, with a threshold of >7, indicating that the data point lay more than seven standard deviations from the mean and was considered an outlier.

#### Feature selection

Feature selection is the process of removing irrelevant or redundant features during the development of a predictive model.^22^ Feature selection methods were applied during data preprocessing to produce more efficient data. Furthermore, The dataset included 48 features, making feature selection essential. Excessive features are time-consuming and resource-intensive, so it is important to speed up model building and improve the model's performance.^23^ As a result, identifying the most important features associated with acting as a contraceptive is a fundamental step. In this study, we employed Recursive Feature Elimination (RFE) to identify the most relevant variables for predicting the utilization of long-acting contraceptives. Since RFE infers the relevance of features by estimating their importance through the algorithm, it selects the most important features.

#### Data transformation

Data transformation involves converting the data into a format suitable for analysis; this may include changing the data's types, scaling, normalizing, and renaming. In this study, we used the one-hot encoding technique to convert string data into integers, ensuring a uniform data type for machine learning classifiers. Before building the model, we also scaled the dataset to standardize it, making it suitable for analysis and improving model training and evaluation.

#### Data discretization

Data discretization is the method of converting continuous data (numerical values) into discrete categories or intervals.^24^ In this study, we employ binning as a technique to enhance the interpretability and performance of classification algorithms by transforming continuous input into categorical input. Data discretization was used to limit the impact of outliers, make analysis easier, and reduce noise by transforming continuous variables into categorical features. For example, the reproductive mother's age is continuous; attributes were discretized into 15–24, 25–34, and 35–49 according to DHS guidelines.^25^

#### Data standardization and data integration

This study uses the “standard Scaler ()” library of “sklearn” to normalize the data to avoid scaling issues for distance-based learning approaches like logistic regression and Gaussian. In this study, we used 8 sub-Saharan African countries' DHS datasets, integrated them based on identification variables, sorted both data files by the identification variables, determined the base (primary) file, and finally merged them using Stata software.

#### Class balancing and hyperparameter tuning

Data preprocessing was performed using the R programming language (v4.5, www.r-project.org), where a rigorous pipeline was implemented to maximize dataset quality. Before model training, the imbalanced dataset was resampled as part of the data preparation process.^26^ To address class imbalance, the Synthetic Minority Oversampling Technique (SMOTE) was applied to the training data. SMOTE generates synthetic minority-class examples by interpolating between existing observations rather than duplicating them, helping prevent machine-learning models from being biased toward the majority class and modestly improving classification performance on imbalanced datasets. Following resampling, hyperparameter tuning was conducted to optimize model performance. Three methods were utilized: Grid Search, which exhaustively evaluates all combinations of predefined hyperparameters; Random Search, which randomly samples hyperparameter combinations from specified ranges for faster exploration; and Bayesian Optimization, a probabilistic approach that iteratively selects promising hyperparameter configurations based on prior evaluations, improving efficiency and predictive performance, particularly in the presence of class imbalance.

#### Model selection

The outcome variable in this study was binary, indicating whether acting contraceptives were utilized (“yes” or “no”). To predict this outcome, twelve machine-learning classifiers were evaluated: Logistic Regression, K-Nearest Neighbors (KNN), Gradient Boosting (GB), Decision Tree, Support Vector Machine (SVM), XGBoost, Neural Network, Gradient Boosting Machine (GBM), Random Forest (RF), AdaBoost, Multi-Layer Perceptron (MLP), and CatBoost. These algorithms were chosen based on previous research demonstrating the effectiveness of machine-learning techniques in classification tasks. Machine learning has been widely applied in reproductive health prediction tasks, and assess maternal risks during pregnancy. The selection of these algorithms was informed by their ease of implementation, interpretability, training efficiency, ability to mitigate overfitting, and speed in predicting unseen data.

#### Association rule mining

Using the Apriori algorithm from R’s *arules* package, we uncovered hidden relationships in the utilization of long-acting contraceptives and their key predictors, transforming raw categorical data into meaningful “if–then” patterns. This method pinpointed which categories of variables most strongly shape LARC utilization, insights that conventional machine learning models often fail to capture. By examining support (frequency of patterns) and confidence (reliability of rules). Each rule followed the structure *X → Y*, where X (antecedent) increased or decreased the likelihood of Y (consequent). The lift statistic clarified the importance of each relationship: a value of 1 indicated no association, values below 1 reflected a negative correlation, and values above 1 showed a positive influence. This straightforward framework transforms complex associations into actionable knowledge about the true drivers of *LARC utilization*.

## Results

### Descriptive results of the background characteristics

Table 1 presents the distribution of the outcome variable by socio-demographic and economic characteristics. Overall, 28.8% of women reported “Yes” and 71.2% “No.” Country-level prevalence varied widely, from 13.4% in Lesotho to 50.9% in Senegal, with higher rates also in Burkina Faso (39.6%) and Kenya (32.3%). “Yes” responses were more common among rural women (31.4%), women with no education (41.7%), poorer women (34.1%), married women (35.9%), and grand multiparous women (39.1%). Women aged 25–49, those with early first sex or birth, and those reporting distance to health facilities as a major problem (31.4%) showed higher prevalence. Overall, the outcome was concentrated among rural, less educated, poorer, married, and multiparous women, with notable country-level differences (Table 1).

**Table 1:** Socio demographic factors in long-acting reversible contraceptive use in Sub-Saharan Africa

Variable	Category	Frequency	Percent
Residence	Urban	13708	47.24%
	Rural	15308	52.76%
Education	No education	5351	18.44%
	Primary	8671	29.88%
	Secondary	12046	41.52%
	Higher	2948	10.16%
LARC usage	No	20672	71.24%
	Yes	8344	28.76%
Age	15–24	8864	30.55%
	25–34	10588	36.49%
	35–49	9564	32.96%
Wealth	Poor	8750	30.16%
	Middle	5795	19.97%
	Rich	14471	49.87%
Marital	Unmarried	14109	48.62%
	Married	14907	51.38%
Distance	Big problem	7964	27.45%
	Not a big problem	21052	72.55%
Parity	Primiparous	10443	35.99%
	Multiparous	13729	47.32%
	Grand multiparous	4844	16.69%
Media	No	9035	31.14%
	Yes	19981	68.86%
First sex	<18	21950	75.65%
	≥18	7066	24.35%
Age at birth	<20	20380	70.24%
	≥20	8636	29.76%
Country	Burkina	3179	10.96%
	Cote d’Ivoire	2090	7.20%
	Ghana	4483	15.45%
	Kenya	5891	20.30%
	Lesotho	3487	12.02%
	Mozambique	4203	14.49%
	Senegal	2595	8.94%
	Tanzania	3088	10.64%

### Pooled prevalence of LARC

The forest plot summarizes the pooled prevalence of long-acting reversible contraceptive (LARC) use across eight African countries. Individual country estimates range from 13% in Lesotho to 51% in Senegal. All country estimates are precise and statistically significant, as indicated by their narrow confidence intervals that do not cross zero. The pooled random-effects estimate shows that approximately 29% (95% CI: 21%–38%) of reproductive-age women use LARC methods overall.

**Figure 2:**
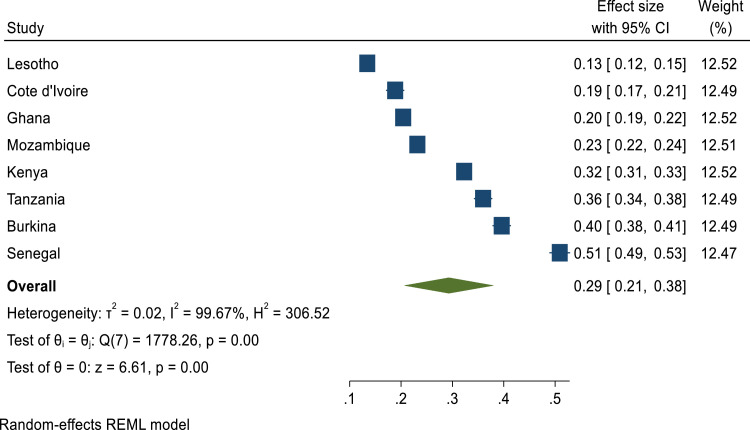
Pooled prevalence of long-acting reversible contraceptive (LARC) utilization across selected African countries

Heterogeneity is extremely high (I²=99.67%, *P*<.001), reflecting substantial variation in LARC uptake between countries. This variation may be due to differences in health-system capacity, access to family planning services, socioeconomic conditions, and policy environments. Despite the heterogeneity, the overall test indicates a significant pooled effect (z=6.61, *P*<.001), suggesting meaningful utilization of LARC across settings, though with wide disparities between countries Figure 2.

Tilahun et al. Determinants and predictive modeling of long-acting reversible contraceptive use in Sub-Saharan Africa. Am J Obstet Gynecol 2026.

### Prediction of utilization of long-acting contraceptives using machine learning algorithms

#### Feature selection

**Figure 3:**
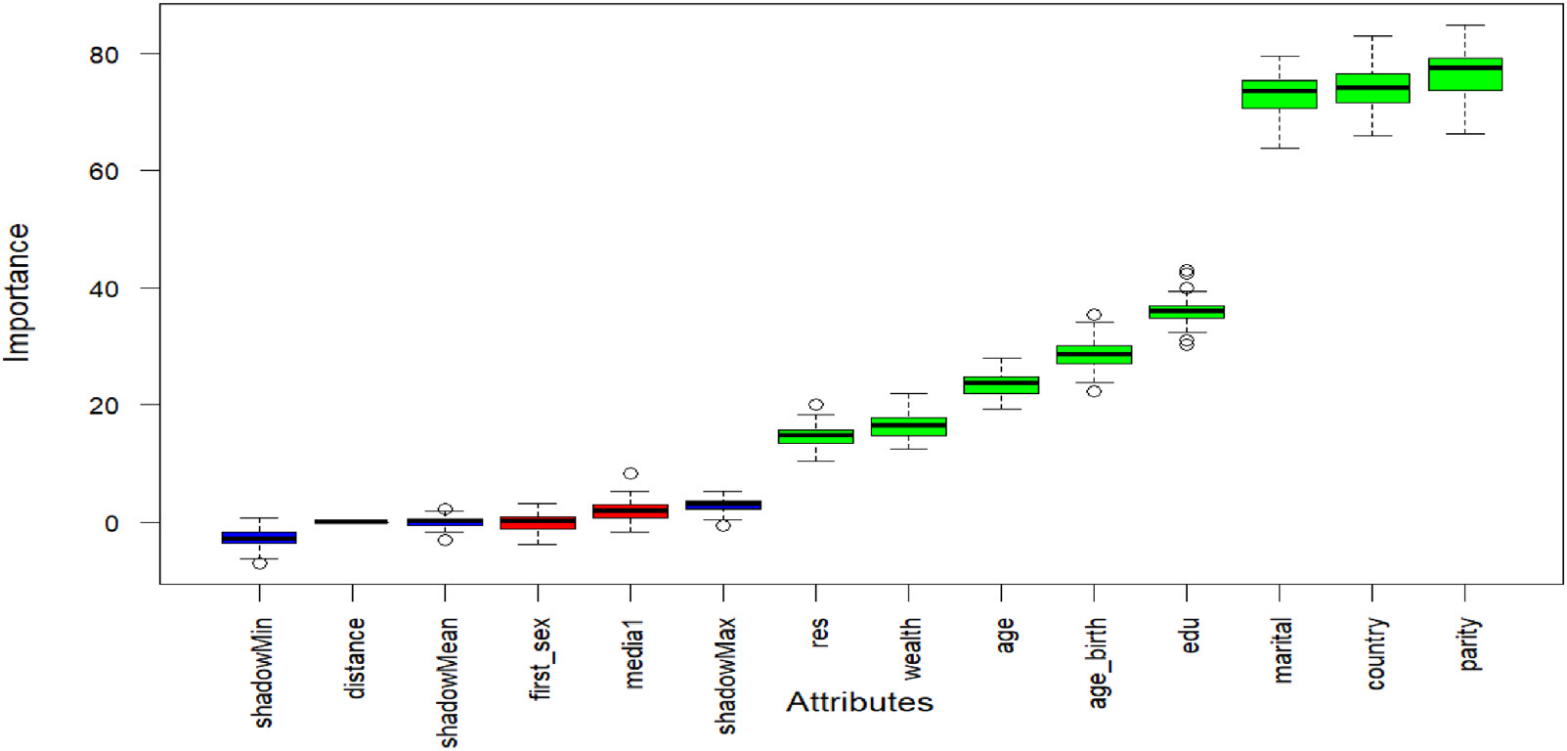
Boruta feature selection for predictors of LARC utilization across selected African countries

The prediction of utilization of long-acting reversible contraceptives using machine learning starts with effective feature selection, accomplished here with the Boruta algorithm. This method systematically evaluates all variables, distinguishing the influential predictors from those with little impact on LARC utilization deoutcomes. It identifies the most critical features that contribute to variations in utilization of long-acting reversible contraceptives, advancing them for further analysis while filtering out weak predictors. The Boruta plot visually categorizes variables: green indicates important features, yellow marks uncertain, and red shows discarded features.^27^ After refining the dataset, only the top-performing features were retained to predict utilization of long-acting contraceptives and reveal hidden patterns through association rule mining (Figure 3).

Tilahun et al. Determinants and predictive modeling of long-acting reversible contraceptive use in Sub-Saharan Africa. Am J Obstet Gynecol 2026.

### Model development and performance evaluation for predicting the utilization of long-acting reversible contraceptives

**Figure 4:**
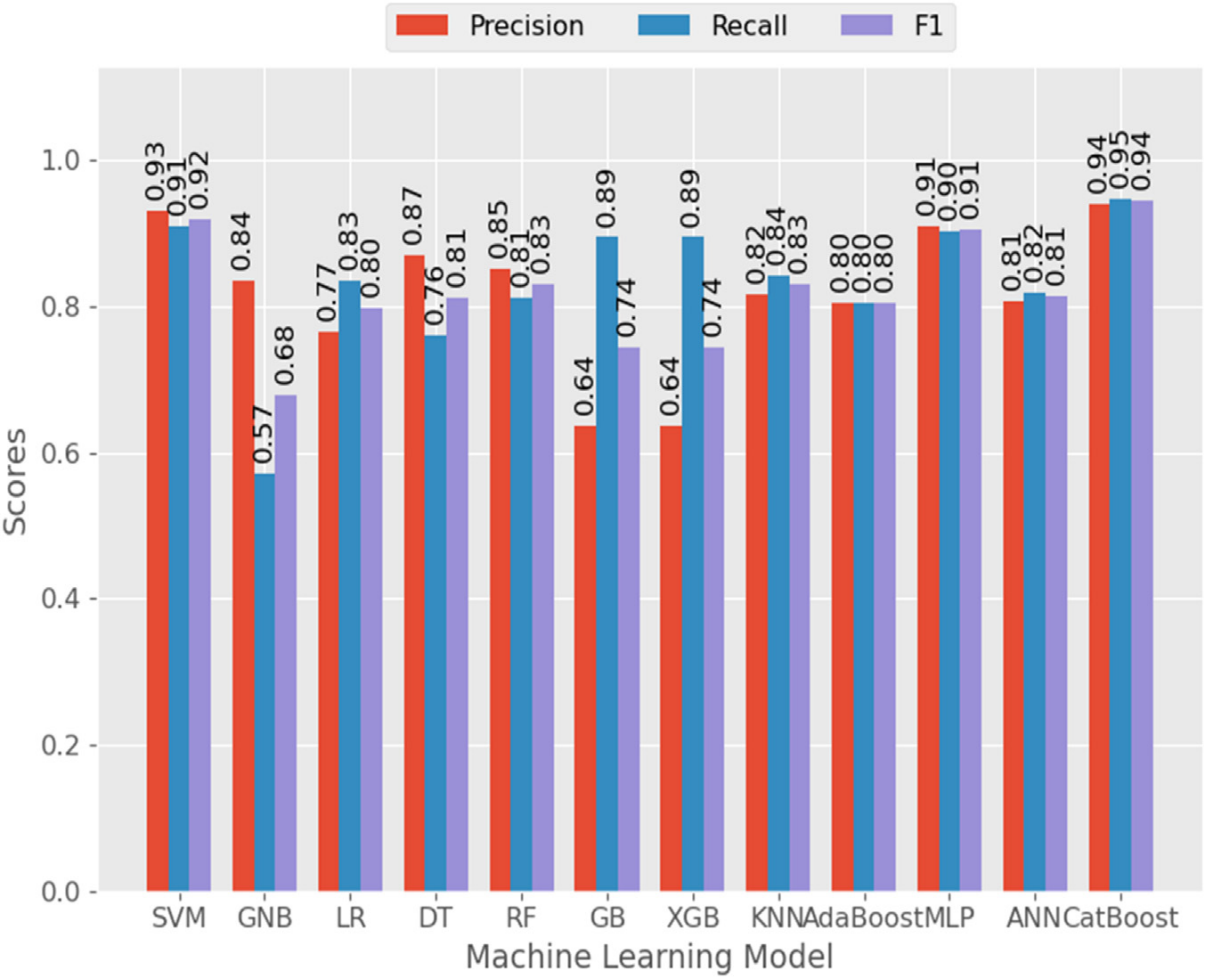
Comparison of precision, recall, and F1-score across machine learning models for predicting long-acting reversible contraceptive (LARC) utilization among reproductive-age women in Sub-Saharan Africa

To ensure optimal predictive accuracy, several machine-learning algorithms were systematically developed, optimized, and compared. Model performance was evaluated using a comprehensive set of metrics, including accuracy, precision, recall, F1 score, specificity, and AUC, which collectively capture overall correctness, the ability to correctly identify true positive and negative cases, and the model’s discriminative capacity. This rigorous assessment enabled the identification of the most effective algorithm for predicting long-acting reversible contraceptive (LARC) utilization among women of reproductive age in sub-Saharan Africa. The comparative results of these models are presented in Supplementary Table 1 and Figure 4, which illustrate the relative performance across optimization methods.

Tilahun et al. Determinants and predictive modeling of long-acting reversible contraceptive use in Sub-Saharan Africa. Am J Obstet Gynecol 2026.

Performance varied notably across the three optimization strategies. Under Grid Search, the Neural Network achieved the highest performance, with an accuracy of 81.3% and an AUC of 79.7%, while Gradient Boosting and Random Forest also showed competitive results. In the Random Search approach, predictive performance further improved Neural Network, Support Vector Machine (SVM), and Random Forest all achieved accuracies above 82%, demonstrating a balanced trade-off across key evaluation metrics. The Bayesian Optimization approach, however, produced the most robust and consistent results, outperforming the other methods. Notably, Random Forest (accuracy =85.%, AUC=81.0%) showed the best overall predictive capability.

Overall, Bayesian Optimization proved to be the most effective tuning strategy, offering superior model stability and predictive power compared to both Grid and Random Search methods (see Figure 3 and Supplementary Table 1). This evidence underscores the reliability and applicability of the final selected models in predicting LARC utilization across diverse population settings.

### Receiver operating characteristic (ROC) curve for the tested models

**Figure 5:**
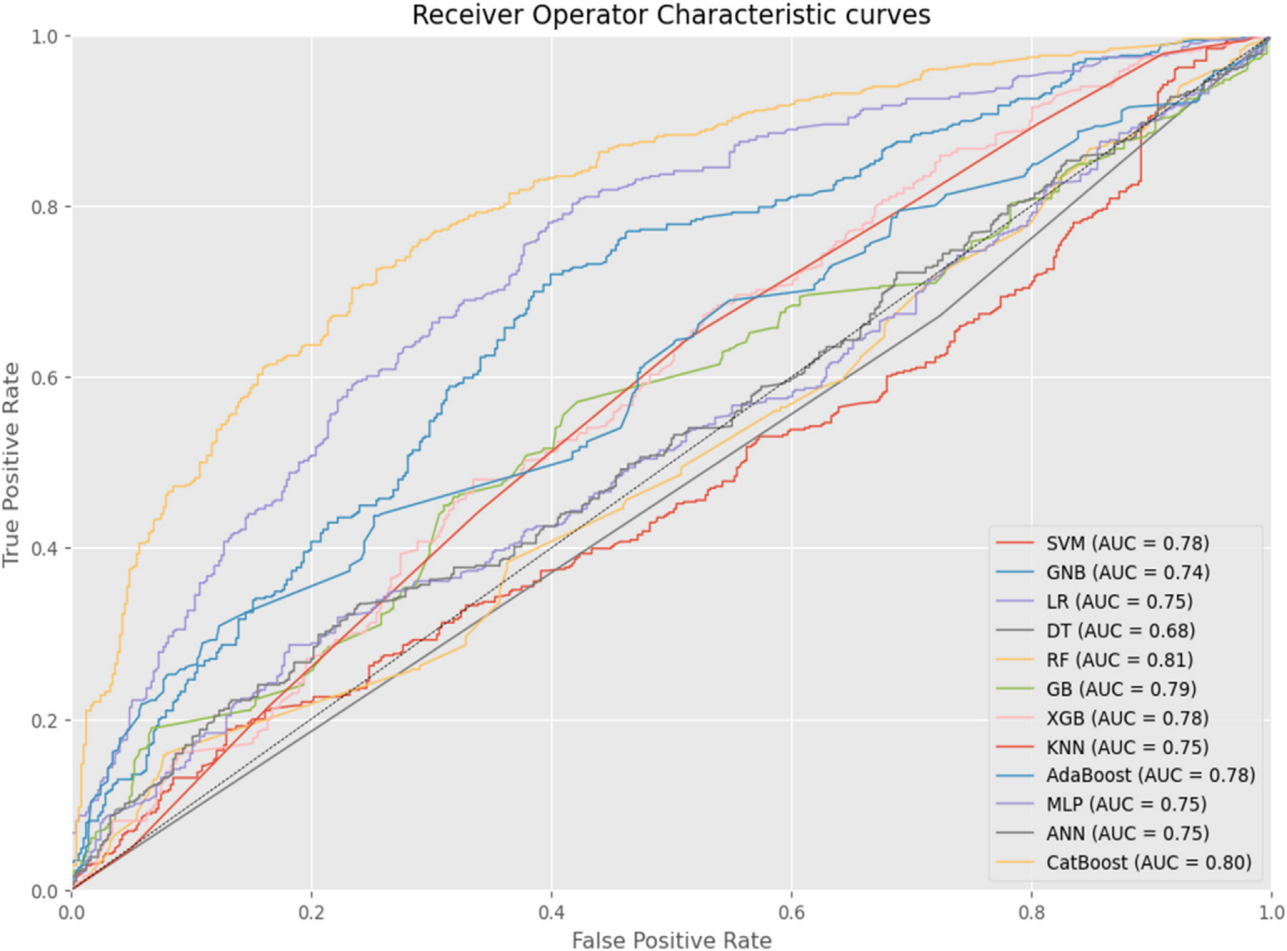
Receiver operating characteristic (ROC) curves comparing the performance of machine learning models in predicting long-acting reversible contraceptive (LARC) utilization among reproductive-age women in Sub-Saharan Africa

The Receiver Operating Characteristic (ROC) curve provides a graphical representation of the classification performance of all tested machine-learning models. The ROC curve plots the true positive rate (sensitivity) against the false positive rate (1 − specificity), thereby illustrating each model’s ability to discriminate between women who utilize long-acting reversible contraceptives (LARC) and those who do not. Among the evaluated algorithms, the Random Forest (RF) model demonstrated the highest Area Under the Curve (AUC) value, indicating superior discriminatory power compared to the other models. Random Forest consistently achieved a strong balance across accuracy, precision, recall, and specificity. The higher AUC value reflects the model’s robustness and reliability in predicting LARC utilization outcomes across sub-Saharan African countries (Figure 5).

Tilahun et al. Determinants and predictive modeling of long-acting reversible contraceptive use in Sub-Saharan Africa. Am J Obstet Gynecol 2026.

### Feature importance

**Figure 6:**
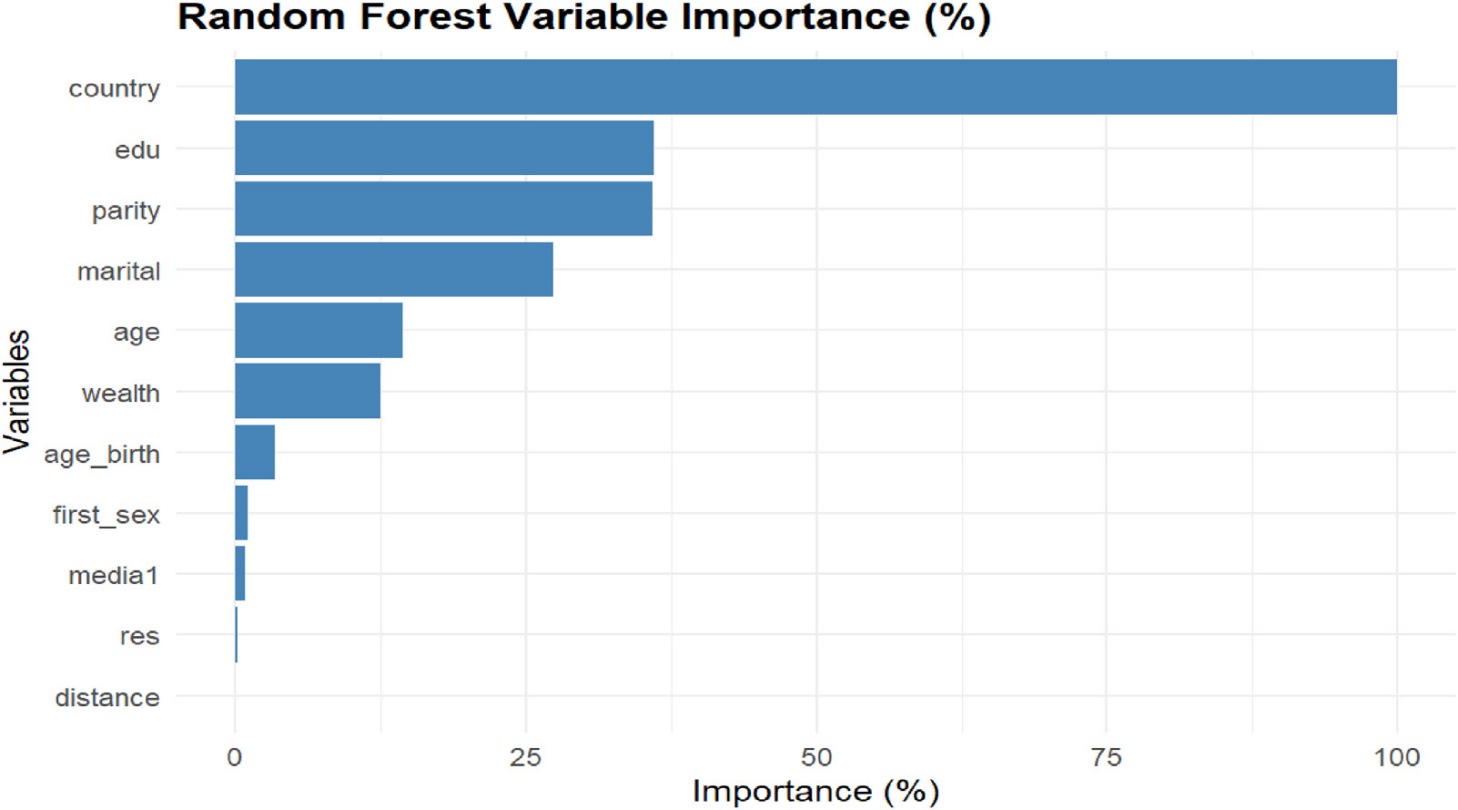
Variable importance plot from the random forest model showing the most influential predictors of long-acting reversible contraceptive (LARC) utilization among reproductive-age women in Sub-Saharan Africa

The results indicate that country is the most influential predictor, contributing nearly 100% importance, followed by education (edu) and parity, which also show strong predictive power. Marital status and age demonstrate moderate influence, whereas variables such as wealth, age at first birth (age_birth), and first sexual intercourse (first_sex) contribute marginally. Factors like media exposure (media1), residence (res), and distance to facility show minimal importance in predicting the outcome. Overall, the model highlights that contextual (country-level) and demographic factors play a more significant role in influencing the outcome than socioeconomic or behavioral variables (Figure 6).

Tilahun et al. Determinants and predictive modeling of long-acting reversible contraceptive use in Sub-Saharan Africa. Am J Obstet Gynecol 2026.

### Association rule mining

Using the Apriori algorithm, we identified eight influential association rules based on lift values and confidence. Notably, variables such as wealth index, women’s educational status, place of residence, parity, age at first birth, and region consistently appeared, demonstrating their strong association with the likelihood of utilizing long-acting reversible contraceptives (LARCs). The top eight association rules and their corresponding lift values are as follows:

1. Women in Senegal who were rural, uneducated, poor, and married had a 64.6% probability of using LARCs. This pattern occurred in 1.21% of cases (*Lift =2.25*, indicating more than twice the baseline likelihood).
2. Women in Senegal who were rural, uneducated, and poor had a 64.1% probability of using LARCs, slightly more common at 1.23% support (*Lift =2.23*).
3. Women in Senegal who were uneducated, poor, and had their first birth before age 20 had a 63.3% probability of LARC use (1.02% support, *Lift =2.20*).
4. Women in Senegal who were uneducated, poor, and married had a 62.8% probability of LARC use (1.39% support, *Lift =2.18*).
5. Rural women in Senegal who were uneducated, married, and had their first birth before age 20 had a 62.4% probability of LARC use (1.08% support, *Lift =2.17*).
6. Rural, married women in Senegal who were grand multiparous had a 62.3% probability of LARC use (1.03% support, *Lift =2.17*).
7. Women in Burkina Faso who were rural, uneducated, married, and had their first birth before age 20 had a 56.6% probability of LARC use (1.25% support, *Lift =1.97*).
8. Rural women who were uneducated, poor, married, grand multiparous, and had their first birth before age 20 had a 53.0% probability of LARC use (1.15% support, *Lift =1.84*).

## Discussion

Using the most recent sub-Saharan Africa Demographic and Health Survey (DHS) data, our study evaluated eight advanced machine-learning algorithms to examine critical reproductive health indicators. Each algorithm was carefully optimized to maximize predictive performance. Our findings reveal a concerning trend: only 28.76% of reproductive-age women in sub-Saharan Africa utilized long-acting contraceptive methods, a figure a bit higher than the 23% previously studied in resource-limited settings.^28^ This persistent rate underscores ongoing challenges in healthcare access and signals stagnation or even a slight decline in facility-based deliveries, highlighting the urgent need for targeted policy interventions. Without swift action, Ethiopia may struggle to meet the Sustainable Development Goals (SDGs) aimed at reducing maternal mortality. Addressing this gap requires innovative strategies, strengthened healthcare systems, and active community engagement to ensure safe childbirth for all women.

Rural residence consistently emerged as an important determinant of LAC utilization. Evidence from Senegal and Burkina Faso indicated that women in rural areas were more likely to adopt LACs than their urban counterparts. Similar findings have been reported in studies conducted in Australia,^29^ the United States,^30^ Nigeria,^31^ Yemen,^27^ and Kenya.^32^ Conversely, research from Indonesia revealed lower utilization rates in rural communities compared to urban ones, while a study from Oregon, United States,^33^ found no significant difference between the two settings. These mixed findings suggest that the association between place of residence and LARC utilization is highly context-specific. In some countries, targeted family planning initiatives, community-based outreach programs, expanded method availability, and donor-supported interventions have improved access to LARCs among underserved and rural populations, thereby reducing traditional geographic barriers to contraceptive services.^34^^,^^35^ Given that country was identified as the most influential predictor in our model, this finding may also reflect differences in national family planning policies, program implementation, and health-system capacity across countries.^34^ Nevertheless, persistent barriers such as distance to health facilities, transportation challenges, and limited service availability continue to pose major obstacles to contraceptive access in many rural settings.^26^

Low educational attainment also emerged as a significant factor. Women without formal education demonstrated a greater likelihood of using LARCs across several rules. Similar patterns have been documented in previous studies.^36^, ^37^, ^38^ This finding may reflect targeted family planning interventions aimed at underserved populations and the convenience of long-acting methods that require minimal follow-up.^34^^,^^39^ However, given the strong influence of country in our model, the observed association may also reflect differences in national family planning programs and service-delivery strategies across countries.^34^ Concerns have also been raised regarding inequities in contraceptive counseling and informed choice among socially disadvantaged women.^40^ Therefore, this finding should be interpreted cautiously. Nonetheless, expanding educational opportunities remains important for empowering women to make informed reproductive health decisions.

Women from poorer households were more likely to use LACs in the observed association rules. This finding is consistent with evidence from other studies, which also reported higher uptake of LACs among women from poorer households.^15^^,^^41^, ^42^, ^43^, ^44^ One possible explanation is that long-acting methods are more cost-effective over time compared to short-term contraceptives, making them attractive for women with limited financial resources.^45^ Additionally, free or subsidized LARC services provided through government and donor-funded family planning programs may disproportionately benefit economically disadvantaged populations.^46^ Targeted outreach initiatives aimed at reducing unmet contraceptive needs among vulnerable populations may also contribute to this pattern.^47^^,^^48^ However, given the strong influence of country in our predictive model, the observed association may partly reflect differences in national family planning policies, program coverage, and service delivery approaches across countries.

Marriage was consistently associated with a higher likelihood of LAC utilization, aligning with findings from other studies.^42^^,^^49^^,^^50^ Married women often have clearer and more predictable fertility intentions, which may encourage the adoption of long-acting methods for both birth spacing and limiting family size. This highlights the importance of integrating marital status into reproductive health programming to improve effectiveness. However, it is equally important to recognize that unmarried women may face distinct barriers to accessing LACs. Social stigma, cultural norms, and provider bias can limit their contraceptive choices, even when their fertility intentions are equally valid. Ignoring this group risks reinforcing inequities in family planning services.^46^^,^^51^^,^^52^ Therefore, interventions should not only leverage the higher uptake among married women but also actively work to reduce barriers and ensure equitable access for unmarried women.

Higher parity, especially grand multiparty, was positively associated with LAC utilization, consistent with findings from other studies.^32^^,^^37^^,^^43^^,^^44^^,^^50^^,^^53^ Women with several children may prefer long-acting methods to avoid additional pregnancies, reflecting a practical response to their accumulated reproductive experiences. This underscores the importance of offering counseling and contraceptive options that are sensitive to parity status. On the other hand, women with lower parity or no children may be less likely to adopt LACs due to concerns about future fertility, misconceptions about reversibility, or societal expectations to have children before using long-term contraception. These barriers highlight the need for counseling that addresses fertility intentions at different parity levels, ensuring that both high- and low-parity women can make informed choices aligned with their reproductive goals.

Early childbearing (before age 20) was associated with a higher likelihood of LARC utilization, consistent with findings from other studies.^36^^,^^37^ Women who begin childbearing at a younger age may face cumulative reproductive risks and health challenges, which can motivate the adoption of long-acting methods as a strategy to space or limit further pregnancies. This highlights the importance of programs that provide adolescents and young women with accurate information on safe spacing and a range of contraceptive options. Conversely, many adolescents and young women encounter barriers to LAC access, including restrictive policies, provider bias, and social stigma around premarital sexual activity. These constraints may prevent those most in need from obtaining effective contraception. Addressing such barriers through youth-friendly, rights-based services is essential to ensure equitable access and reduce unintended pregnancies among this group.

The association rules underscored that national and regional contexts shape LAC utilization patterns. For instance, findings from Senegal differed somewhat from those of Burkina Faso, which aligns with evidence showing that Senegal had one of the lowest family planning utilization rates globally at 26% in 2017.^54^ This may be influenced by cultural norms, health system capacity, programmatic reach, and the relatively high discontinuation rate of intrauterine devices (18.4%) reported in Senegal.^55^ Such variations highlight the importance of tailoring interventions to the local context in order to strengthen contraceptive coverage and improve effectiveness.

Importantly, these determinants seldom operated in isolation. The association rules showed that intersecting factors, such as rural residence, low educational attainment, poverty, and marital status, collectively increased the likelihood of LARC utilization. This indicates that contraceptive behavior is shaped by overlapping social and demographic vulnerabilities rather than single predictors. Consequently, multidimensional strategies that simultaneously address structural, educational, and socioeconomic barriers are critical for strengthening contraceptive uptake.

At the same time, such intersections can also exacerbate inequities, as some women may adopt LARCs out of limited alternatives rather than genuine choice. Ensuring that utilization reflects informed, voluntary decision-making requires that programs integrate rights-based approaches alongside efforts to expand access.

### Strengths and limitations of the study

This study has several notable strengths. First, the use of large, multicountry, and nationally representative Demographic and Health Survey (DHS) data enhances the generalizability of the findings across diverse populations in Sub-Saharan Africa. The inclusion of multiple countries captures a wide range of socioeconomic, cultural, and health system contexts, providing a comprehensive understanding of long-acting reversible contraceptive (LARC) utilization patterns. Second, the integration of advanced machine learning methods, specifically Random Forest, SHAP (Shapley Additive Explanations), and the Apriori algorithm, improves both predictive accuracy and interpretability. These modern analytical tools allow for a deeper exploration of complex, nonlinear relationships among determinants of contraceptive use. Third, rigorous data preprocessing, including Synthetic Minority Over-Sampling Technique (SMOTE) for class balancing and Recursive Feature Elimination (RFE) for variable selection, ensured robust model training and reduced bias arising from imbalanced data.

Despite these strengths, the study has some limitations that should be acknowledged. The cross-sectional nature of DHS data limits the ability to draw causal inferences between predictor variables and LARC utilization. In addition, reliance on self-reported measures introduces potential recall and reporting biases, particularly concerning sensitive reproductive health information. Moreover, variations in national policies, program implementation, and health service accessibility across countries were not fully captured, which may have influenced observed differences in contraceptive use. Lastly, while machine-learning models effectively identify key predictors, they may not fully account for unmeasured contextual factors such as cultural norms, provider attitudes, or policy enforcement variations that influence women’s contraceptive choices.

## Conclusion and recommendation

In conclusion, this study demonstrated that machine-learning techniques can effectively predict and explain the utilization of long-acting reversible contraceptives (LARCs) among women of reproductive age in Sub-Saharan Africa. Despite moderate progress, significant disparities persist across education, residence, and socioeconomic status, indicating the need for context-specific strategies. The Random Forest and SHAP analyses revealed that education, marital status, parity, and place of residence are the most influential determinants of LARC use. To address these gaps, targeted interventions should prioritize rural areas and women with limited education, while strengthening community-based awareness and service delivery. Integrating LARC counseling into maternal and child health programs and applying data-driven policy approaches using predictive analytics can enhance efficiency and equity. Policymakers should ensure that family planning services are inclusive, accessible, and responsive to women’s diverse needs, particularly those from poor and remote communities, to improve reproductive health outcomes and achieve universal access to modern contraception.

## Availability of data and materials

Additional data can be available from the corresponding author upon reasonable request.

## CRediT authorship contribution statement

**Befkad Derese Tilahun:** Writing – review & editing, Writing – original draft, Validation, Supervision, Resources, Investigation, Formal analysis, Conceptualization. **Mulat Ayele:** Writing – review & editing, Writing – original draft, Visualization, Software, Funding acquisition, Formal analysis, Data curation, Conceptualization. **Eyob Shitie Lake:** Writing – review & editing, Writing – original draft, Validation, Resources, Methodology, Investigation, Data curation. **Gizachew Yilak:** Writing – review & editing, Writing – original draft, Visualization, Validation, Software, Investigation, Formal analysis, Conceptualization. **Tegene Atamenta Kitaw:** Writing – review & editing, Writing – original draft, Visualization, Validation, Supervision, Methodology, Investigation, Formal analysis. **Bekalu Alemayehu Attinafu:** Writing – review & editing, Writing – original draft, Validation, Resources, Investigation, Data curation, Conceptualization.

## Appendix Supplementary materials

Image, application 1

## Footnotes

Supplementary material associated with this article can be found in the online version at doi:10.1016/j.xagr.2026.100662.
