# Supplementary material for: Determinants and predictive modeling of long-acting reversible contraceptive use in Sub-Saharan Africa: evidence from DHS data using machine learning and association rule mining
Source: AJOG Glob Rep. 2026 Jun 12;6(3):100662. doi: 10.1016/j.xagr.2026.100662 (PMC13356621; doi:10.1016/j.xagr.2026.100662)
Supplement: Supplementary file 1 [file mmc1.docx]

Supplementary Table 1. Performance Comparison of Machine Learning Models for Predicting utilization of long-acting contraceptives Across Different Scenarios (Grid search, Random search, and Baysian optimization)

| Scenario | Algorithm | AUC | Accuracy | Precision | F1 Score | Sensitivity | Specificity |
| --- | --- | --- | --- | --- | --- | --- | --- |
| Grid search | Logistic Regression | 77.5 | 77.5 | 54.3 | 53.6 | 81.7 | 72.9 |
|  | KNN | 75.6 | 76.0 | 83.2 | 81.2 | 75.3 | 76.0 |
|  | GB | 76.2 | 78.6 | 85.1 | 84.0 | 83.3 | 83.3 |
|  | Decision Tree | 72.3 | 75.7 | 82.4 | 84.6 | 81.1 | 82.6 |
|  | SVM | 73.9 | 71.5 | 84.1 | 79.5 | 71.9 | 70.4 |
|  | XGBoost | 78.8 | 78.3 | 55.6 | 54.3 | 53.1 | 88.7 |
|  | Neural Network | 79.7 | 81.3 | 51.1 | 58.4 | 68.7 | 75.2 |
|  | GBM | 70.1 | 78.5 | 56.3 | 56.6 | 87.5 | 76.9 |
|  | Random Forest (RF) | 77.8 | 76.4 | 83.8 | 86.1 | 85.4 | 74.7 |
|  | AdaBoost | 73.5 | 73.8 | 74.0 | 73.5 | 75.0 | 72.5 |
|  | MLP | 74.2 | 75.5 | 78.0 | 77.0 | 79.0 | 74.0 |
|  | CatBoost | 75.3 | 76.2 | 81.0 | 80.5 | 82.0 | 77.0 |
| Random search | Logistic Regression | 79.2 | 71.4 | 76.7 | 86.0 | 79.0 | 75.6 |
|  | KNN | 79.1 | 81.6 | 84.1 | 87.4 | 85.0 | 67.6 |
|  | GB | 77.9 | 76.2 | 84.1 | 85.1 | 80.0 | 83.4 |
|  | Decision Tree | 60.0 | 81.2 | 81.2 | 83.2 | 85.0 | 80.0 |
|  | SVM | 72.5 | 82.0 | 83.1 | 88.0 | 83.1 | 82.4 |
|  | XGBoost | 79.1 | 81.3 | 84.1 | 87.0 | 85.0 | 80.9 |
|  | Neural Network | 79.0 | 82.3 | 84.0 | 87.8 | 85.0 | 89.6 |
|  | GBM | 71.8 | 82.2 | 83.6 | 87.9 | 85.0 | 75.6 |
|  | Random Forest (RF) | 76.9 | 82.0 | 83.7 | 87.7 | 85.0 | 76.5 |
|  | AdaBoost | 75.5 | 76.7 | 80.0 | 82.0 | 80.0 | 74.0 |
|  | MLP | 77.0 | 78.2 | 82.5 | 79.0 | 81.0 | 77.5 |
|  | CatBoost | 78.0 | 79.5 | 80.0 | 81.0 | 83.0 | 80.0 |
| Baysian optimization | Logistic Regression | 74.2 | 81.7 | 77.0 | 80.0 | 75.0 | 81.0 |
|  | KNN | 79.2 | 84.2 | 82.0 | 83.0 | 80.0 | 79.6 |
|  | GB | 73.0 | 83.3 | 64.0 | 84.0 | 84.0 | 81.1 |
|  | Decision Tree | 79.8 | 82.9 | 76.0 | 81.0 | 85.8 | 73.4 |
|  | SVM | 73.8 | 83.4 | 88.0 | 87.0 | 88.6 | 75.4 |
|  | XGBoost | 81.9 | 77.0 | 64.0 | 74.0 | 81.0 | 76.5 |
|  | Neural Network | 79.8 | 81.5 | 81.0 | 81.0 | 81.75 | 82.5 |
|  | GBM | 76.3 | 90.0 | 89.0 | 68.0 | 85.0 | 83.0 |
|  | Random Forest (RF) | 81.0 | 87.1 | 81.0 | 85.0 | 87.9 | 88.9 |
|  | AdaBoost | 78.5 | 78.0 | 75.0 | 77.0 | 81.0 | 79.0 |
|  | MLP | 78.0 | 81.0 | 79.0 | 80.0 | 81.0 | 82.0 |
|  | CatBoost | 79.8 | 82.5 | 82.0 | 83.0 | 83.0 | 80.0 |
